# Assessment of suicide risk in stabilized bipolar patients

**DOI:** 10.1192/j.eurpsy.2025.1039

**Published:** 2025-08-26

**Authors:** M. Abdelkefi, S. Ellouze, I. Chaari, N. Bouattour, R. Jbir, N. Halouani, M. Turki, J. Aloulou

**Affiliations:** 1 Psychiatry B, Hedi Chaker university hospital, Sfax, Tunisia

## Abstract

**Introduction:**

Suicide risk is a significant concern in bipolar disorder, with a notably higher rate of suicidal behaviors compared to the general population. Stabilized bipolar patients, while in remission, remain at risk due to the chronic nature of the illness and its associated mood dysregulation.

**Objectives:**

This study aims to evaluate the prevalence and characteristics of suicide risk in a sample of stabilized bipolar patients.

**Methods:**

We approached 107 stabilized bipolar patients attending the psychiatry outpatient unit at the Hedi Chaker University Hospital in Sfax. Ninety-three patients agreed to participate in the study. We collected their sociodemographic and clinical data. Suicide risk was assessed using the Mini International Neuropsychiatric Interview (MINI).

**Results:**

The mean age of the participants was 41.49±12.33 years, with a predominance of males (72%). Among the patients, 58.1% were married, 47.3% were unemployed, and 44.1% reported low income. Medical comorbidities were reported by 35.5% of patients, while 11.8% had psychiatric comorbidities in addition to bipolar disorder.

Lifestyle factors revealed that 49.5% of the participants were smokers, 11.8% consumed alcohol, and 2.2% used cannabis.

Most of the patients were diagnosed with type I BD (74.2%), and 18 patients (19.4%) had a history of attempted suicide.

At the time of the study, 19.4% of the patients were assessed as being at risk of suicide with 17.2% presenting low risk and 2.2% exhibiting moderate risk.

**Conclusions:**

This study reveals that a significant portion of stabilized bipolar patients remain at risk for suicide, with nearly one in five participants showing some level of suicide risk despite their clinical stabilization. While most were categorized as low risk, the findings underscore the necessity for continuous suicide risk assessments and preventive strategies, even during periods of mood stability.

**Disclosure of Interest:**

None Declared

